# Effects of tomato inoculation with the entomopathogenic fungus *Metarhizium brunneum* on spider mite resistance and the rhizosphere microbial community

**DOI:** 10.3389/fmicb.2023.1197770

**Published:** 2023-05-24

**Authors:** Shumaila Rasool, Andreas Markou, S. Emilia Hannula, Arjen Biere

**Affiliations:** ^1^Department of Terrestrial Ecology, Netherlands Institute of Ecology (NIOO-KNAW), Wageningen, Netherlands; ^2^Institute of Environmental Sciences, Leiden University, Leiden, Netherlands

**Keywords:** entomopathogenic fungi, *Metarhizium*, spider mites, metabolites, rhizosphere microbial communities

## Abstract

Entomopathogenic fungi have been well exploited as biocontrol agents that can kill insects through direct contact. However, recent research has shown that they can also play an important role as plant endophytes, stimulating plant growth, and indirectly suppressing pest populations. In this study, we examined the indirect, plant-mediated, effects of a strain of entomopathogenic fungus, *Metarhizium brunneum* on plant growth and population growth of two-spotted spider mites (*Tetranychus urticae*) in tomato, using different inoculation methods (seed treatment, soil drenching and a combination of both). Furthermore, we investigated changes in tomato leaf metabolites (sugars and phenolics), and rhizosphere microbial communities in response to *M. brunneum* inoculation and spider mite feeding. A significant reduction in spider mite population growth was observed in response to *M. brunneum* inoculation. The reduction was strongest when the inoculum was supplied both as seed treatment and soil drench. This combination treatment also yielded the highest shoot and root biomass in both spider mite-infested and non-infested plants, while spider mite infestation increased shoot but reduced root biomass. Fungal treatments did not consistently affect leaf chlorogenic acid and rutin concentrations, but *M. brunneum* inoculation via a combination of seed treatment and soil drenching reinforced chlorogenic acid (CGA) induction in response to spider mites and under these conditions the strongest spider mite resistance was observed. However, it is unclear whether the *M. brunneum*-induced increase in CGA contributed to the observed spider mite resistance, as no general association between CGA levels and spider mite resistance was observed. Spider mite infestation resulted in up to two-fold increase in leaf sucrose concentrations and a three to five-fold increase in glucose and fructose concentrations, but these concentrations were not affected by fungal inoculation. *Metarhizium*, especially when applied as soil drench, impacted the fungal community composition but not the bacterial community composition which was only affected by the presence of spider mites. Our results suggest that in addition to directly killing spider mites, *M. brunneum* can indirectly suppress spider mite populations on tomato, although the underlying mechanism has not yet been resolved, and can also affect the composition of the soil microbial community.

## 1. Introduction

In recent years, national and international regulations have banned the use of many pesticides for pest management due to environmental and health concerns (Agut et al., 2018). This has created a need for the development and optimization of more sustainable pest control strategies. Entomopathogenic fungi (EPF) have been widely recognized as microbial agents for controlling arthropod pests. They can directly interact with a broad range of arthropod pests, causing high mortality rates through mycosis. However, many EPF can also colonize plants and recently there has been a growing interest in their roles as endosymbionts of plants that can not only improve plant health by increasing nutrient and water uptake but also indirectly enhance plant resistance to insect herbivores by inducing or priming plants for enhanced defenses (Vega et al., 2009; Behie and Bidochka, 2014a). Plant-beneficial microbes including EPF have been shown to affect plant-insect interactions at the individual and community levels (Hartley and Gange, 2009; Simon et al., 2017). In these multifaceted interactions, microbes influence insect herbivores feeding on the host plant and, conversely, insects may affect microbial communities, through plant-mediated effects (Biere and Bennett, 2013).

*Metarhizium* (Clavicipitaceae) is the most abundant and diverse genus of EPF, harboring species with direct pathogenic effects on a broad range of arthropod pests (St. Leger and Wang, 2020). As plant endophytes, different *Metarhizium* spp. have been shown to exert negative effects on arthropod pests including pestiferous spider mites (Canassa et al., 2020; Rasool et al., 2021a). However, strains of *Metarhizium brunneum* with great potential to cause direct insect virulence have shown divergent indirect effects as plant endophytes, ranging from positive to negative impacts on pest populations (Clifton et al., 2018; Ment et al., 2020). This is in line with the observation that plant-mediated effects of fungal endophytes on arthropod pests, in general, are variable (Gange et al., 2019) and highlights the need for additional research to thoroughly comprehend the mechanisms underlying the indirect impacts of EPF endophytes on plant resistance.

Symbiotic or mutualistic associations with microbes can affect plant responses to arthropod pests through modulation of metabolite profiles of plants, or plant defense signaling cascades that are triggered after the perception of herbivore attack (Kim and Felton, 2013; Cachapa et al., 2021). Inoculations with entomopathogens can result in the production of plant secondary metabolites involved in defense responses including alkaloids, phenolics and flavonoids (Mantzoukas and Eliopoulos, 2020). Indeed, recent studies have shown that the effects on the population growth of aphids and spider mites were associated with changed profiles of plant secondary metabolites in *Beauveria* and *Metarhizium-*inoculated plants (Rasool et al., 2021a,b). In addition, primary metabolites such as plant-derived sugars have been shown to play a key role in plant-herbivore as well as plant-fungus relationships (Dai et al., 2021). Due to their role as energy sources, these sugars also influence plant growth and development and play a role in defense responses (Ramon et al., 2008; Morkunas and Ratajczak, 2014; Jeandet et al., 2022). However, in general, the involvement of more specialized metabolites in plant-fungus-herbivore interactions and the role of different inoculation methods on these responses are largely unknown.

The method of inoculation is a crucial determinant of the efficacy and colonization pattern of EPF on host plants. Foliar applications tend to promote leaf colonization, while seed treatments or soil drenching favor root and stem colonization (Bamisile et al., 2018). Additionally, the extent of growth-promoting effects of EPF varies with the inoculation method used (Raya-Díaz et al., 2017; Afandhi et al., 2019). Although seed treatment and soil drenching are the commonly employed methods, the impact of different colonization patterns and frequencies resulting from these methods on the physiological and ecological responses of the host plant and its interactions with aboveground herbivores have yet to be investigated.

Plants provide a favorable environment to soil microbial communities in their rhizosphere, including EPF, by the secretion of root exudates (Bruck, 2010). The rhizosphere serves as the primary environment for soil-borne EPF (Hu and St. Leger, 2002). The functions provided by soil micro-biota are fundamental to the soil and plants. One of the concerns of microbial inoculations is that the application of high concentrations of bio-inoculants, including fungi and bacteria, can cause unintended effects on the diversity and composition of soil microbial communities that ultimately influence plant performance and interaction with other organisms (Trabelsi and Mhamdi, 2013; Cornell et al., 2021). It is therefore interesting to see how different EPF inoculation methods with low (seed treatment) and high (soil drenching and combination) fungal load affect the diversity and composition of the rhizosphere soil microbial community.

In this study, we examined the indirect, plant-mediated, effects of *M. brunneum* on plant growth and population growth of two-spotted spider mites in tomato, using different inoculation methods (seed treatment, soil drenching, and a combination of both). The two-spotted spider mite (*Tetranychus urticae* Koch) is one of the most striking examples of a cosmopolitan and polyphagous pest worldwide (Migeon et al., 2010). This fast-reproducing, piercing-sucking herbivore ravages crop value through voracious feeding and the production of toxins, webs and feces at feeding sites (Attia et al., 2013). The possession of a unique set of evolving digestion and detoxification genes makes spider mites unmatched among arthropod herbivores in the development of pesticide resistance (Grbić et al., 2011). Furthermore, we investigated the potential role of a pre-selected set of leaf metabolites in mediating effects of fungal inoculation on spider mites. Specifically, we analyzed two phenolic compounds (chlorogenic acid and rutin), sugars, and leaf C:N ratio in response to fungal inoculation and spider mite feeding. Compounds such as chlorogenic acids and rutin constitute more than half of the total amount of phenolics in tomato leaf tissues (Hoffland et al., 2000) and have been shown to incur a broad spectrum of anti-pathogenic and anti-herbivory activities (Yang et al., 2016; Martínez et al., 2017; Kundu and Vadassery, 2019). Finally, the effect of different inoculation methods with low (seed treatment) and high (soil drenching and combination) fungal load and spider mite feeding was assessed on the diversity and composition of the rhizosphere soil microbial community.

## 2. Materials and methods

### 2.1. Study organisms: plant, fungus, and herbivore

Untreated seeds of *Solanum lycopersicum* (cv. Moneymaker from Oranjeband zaden, The Netherlands) were surface sterilized by soaking in 70% ethanol for 1 min and 1% sodium hypochlorite (NaClO) for 10 min followed by three rinses with sterile double-distilled water. Seeds were air-dried for 30 min under sterile conditions. Surface sterilization success was checked by plating 100 μl water from the last rinse on Sabouraud dextrose agar (SDA) medium and incubated for 10 days in darkness at 23°C. No sign of contamination was observed.

*Metarhizium brunneum* strain 1868, originally isolated from an *Agriotes* sp. adult (provided by the Agricultural Institute of Slovenia) was selected based on its endophytic colonization abilities and high direct pathogenicity against insect pests (Razinger et al., 2020). The fungal isolate was stored at −80°C in the mycological collection at the Netherlands Institute of Ecology, The Netherlands. For the experiments, the fungus was propagated on SDA media supplemented with yeast extract and incubated for 14 days at 24 ± 1°C in darkness.

*Tetranychus urticae* were obtained from a rearing colony established at Wageningen University (Wageningen, The Netherlands) and maintained on tomato plants for several generations in a growth chamber (25 ± 2°C, 16:8 LD and 60–70% RH). For the experiment described below, around 150 adult females were collected from the rearing colony and placed on a fresh young tomato plant for egg-laying. After 24 h the adult mites were removed and the plant with eggs was maintained under the same conditions for 16 days to obtain synchronized-age adult mites. From this cohort, adult females (recognized by large size oval-shaped bodies) were used in the experiment.

### 2.2. *M. brunneum* suspension

Fungal suspensions were prepared by harvesting conidia from sporulating cultures with a sterilized spatula. Conidia were suspended in 0.01% Triton X-100, vortexed and filtered through several layers of sterile cheesecloth to remove hyphal debris and media remnants (Keyser et al., 2014). Fungal concentrations were adjusted to 1 × 10^8^ conidia ml^–1^ using a Fuchs-Rosenthal hemocytometer (0.0625 mm^2^, depth 0.200 mm) under the microscope. Fungal viability was tested by propagating 100 μl of 1 × 10^5^ conidia ml^–1^ dilution on three SDA plates for 24 h at 23°C. Spore germination was checked by counting germinated (if germ tube was twice the length of the conidium) and non-germinated spores on around 1.5 cm^2^ agar piece under the microscope. The isolate showed >90% viability.

### 2.3. Experimental setup

A greenhouse experiment was conducted at the Netherlands Institute of Ecology, Wageningen, the Netherlands during the spring of 2022. The experiment followed a factorial design with four fungal treatments (seed treatment–ST, soil drenching–SD, a combination of seed treatment and soil drenching–STSD, and no fungus control–C) and two herbivory treatments (with or without spider mites). Batches of 80 sterilized seeds were added to 250 ml blue-capped glass bottles with 30 ml of solution, either a suspension of 1 × 10^8^ conidia ml^–1^ of *M. brunneum* (ST and STSD treatments) or 0.01% Triton X-100 (SD and C treatments) and agitated at 100 rpm for 24 h. Seeds either treated with *M. brunneum* or Triton X-100 were individually sown in separate nursery trays containing a soil-sand mixture in the greenhouse (25°C for 16 h day and 18°C for 8 h night with 50–60% RH). The soil was a non-sterile (live), low nutrient, natural sandy soil collected from an arable field in the vicinity of Wageningen (The Netherlands), homogenized by sieving through a 5 mm sieve, and mixed (3:1, w/w) with coarse sand (0.71–1.25 mm from Wildkamp, Lutten, The Netherlands).

Two weeks after sowing, 26 uniform seedlings per inoculation treatment were transferred to 1 L pots (11 × 11 × 12 cm) containing the same soil-sand mix substrate (800 g/pot). Each pot was placed on an individual plastic saucer (φ 16 cm) to prevent cross-contamination. One day after transplantation, 2 ml of the 1 × 10^8^ conidia ml^–1^ suspension of *M. brunneum* was added to the soil around the base of seedlings for the SD and STSD treatments, whereas 2 ml of 0.01% Triton X-100 was applied to the soil around seedlings allocated to the ST and C treatments. After inoculations, the soil surface was covered with a thin layer of sand to avoid algal growth. In total, 104 pots representing four inoculation treatments (ST, SD, STSD, and C) were placed on 10 carts (each representing one block) following a completely randomized block design. Plants were irrigated every second day and fertilized with 1/2 Hoagland nutrient solution once a week through the saucers. Carts were rotated twice a week to account for climatic variations in the greenhouse. Four weeks after sowing, six of the 26 plants per treatment were harvested (Timepoint 1, T1, details below). The remaining 20 plants per treatment were used for a *T. urticae* bioassay (described below), initiated immediately after T1, and harvested 6 weeks after sowing (Timepoint 2, T2) (Supplementary Figure 1).

### 2.4. *T. urticae* bioassay

Fourteen of the twenty plants per treatment from T2 were infested with spider mites, whereas the remaining six received no spider mites and served as spider mite controls. Five adult female spider mites were released on three terminal leaflets of the second oldest true leaf of plants assigned to the spider mite treatment. To prevent spider mites from escaping, a foam band was placed around the stem at the base of the infested leaf. The band was also placed at the same location on non-infested plants. One day after infestation, mites were counted to check the number of mites that were successfully established on infested plants. The numbers of juvenile and adult spider mites were recorded at 3, 7, and 14 days after infestation using a handheld magnifier. After the last count, spider mites were removed using a fine paintbrush. In addition to the total number of spider mites, the number of spider mites produced per successfully established female was calculated by dividing the total number of mites at day 14 by the initial number of successfully established females after 24 h of infestation.

### 2.5. Data collection and sampling

To evaluate the effects of the four fungal treatments on plant biomass production and rhizosphere microbial community composition, plants were harvested 4 weeks (T1, *n* = 6 per fungal treatment) and 6 weeks (T2, *n* = 20 per fungal treatment, 14 with and 6 without spider mites) after sowing. From each plant, three terminal leaflets from the 2nd oldest true leaf were harvested with scissors, placed in a 15 ml falcon tube and immediately flash-frozen in liquid nitrogen for leaf chemical analysis. The scissors were sterilized with ethanol between the plants. For the rhizosphere soil samples, 18 replicates per fungal treatment were used (all 6 from T1, all 6 non-infested plants from T2, and 6 randomly selected replicates from the fourteen infested plants from T2). Bulk soil was removed from the roots by shaking. The remaining soil adjacent to the roots was gently removed with a brush on a sterilized surface and put in a 2 ml Eppendorf tube. Samples for chemical and rhizosphere microbial community analysis were kept at −80°C until further processing. Aboveground plant material from all plants (leaves and stem) was harvested and roots were thoroughly washed under running tap water to remove any remaining soil and dried on tissue papers to remove excess water. Fresh biomass of root and shoot was recorded on an electronic balance. After fresh weight measurements, stem and root tissues were subsampled for endophytic colonization check (details below), and the tissues were weighed again and transferred to paper bags for drying. The root and shoot tissues were dried at 65°C for 2 days and the dry biomass was recorded using the same electronic balance.

### 2.6. Isolation of endophytic fungi

Endophytic colonization of all tomato plants was checked at T1 and T2. From each plant, three root and three stem sections (approx. 3 cm pieces from the base, middle, and upper parts) were harvested. Samples of lower, middle, and upper leaves from a random subset of three plants per treatment were collected for endophytic check in leaves. Stem and leaf sections were included to assess whether in addition to roots also aboveground tissues of tomato can be colonized by this *M. brunneum* strain following the different inoculation methods (Rasool et al., 2021a). The cut plant parts were individually surface sterilized by dipping in 70% ethanol and 2% NaClO for 2 min followed by three rinses with sterilized water. The outer edges of the sterilized pieces were excised with a sterile scalpel to eliminate the tissue parts that had come into contact with the disinfection solution and the pieces were further divided into two parts (1–1.5 cm each), resulting in six roots and six stem parts per plant. All 12 pieces from one plant were placed on a single selective media plate prepared according to Rasool et al. (2021a). Leaf samples were cut into 2 cm^2^ sections and placed on different plates. The pieces were gently pressed into the media with forceps to ensure contact, closed with parafilm, and incubated for 21 days in darkness at 23–24°C. Fungal outgrowth was recorded every 5 days. Sterilization efficacy was checked by plating 100 μl of the last rinse after sterilization on SDA media (Tall and Meyling, 2018). No contamination was observed after sterilization of the stem and leaves, however, in a few cases primary root sections showed random microbial outgrowth. The identification of endophytic fungi growing from edges was done by colony morphology and conidial structures of *M. brunneum* (Humber, 1997; Bich et al., 2021). The *Metarhizium* outgrowth from plant tissue pieces was recorded as a value of one, while no outgrowth was recorded as a value of zero. Endophytic outgrowth of some unidentified fungi was also observed, which was not considered for this experiment.

### 2.7. Chemical analysis

#### 2.7.1. Preparation of plant extracts

The leaf samples were extracted for the analysis of polyphenolics and sugars following the methodology proposed by Pineda et al. (2020). Briefly, 1 ml of 70% methanol (gradient grade for HPLC) was added to 50 mg of ground leaf sample, vortexed, subjected to a sonicator for 30 min (20°C), and centrifuged at 10,000 rpm for 10 min. The supernatant was collected and the procedure was repeated to increase extraction efficiency with a 2 ml final volume. The extracts were filtered through a 0.2 μm polytetrafluoroethylene syringe filter (Henske Sass Wolf GmbH, Tuttlingen, Germany) and stored at −20°C until analysis. The same extracts were used for polyphenolics and sugar analysis.

#### 2.7.2. Quantification

Analysis of phenolic compounds (chlorogenic acid and rutin) was performed by high-performance liquid chromatography (HPLC, ThermoFisher Scientific, Waltham, MA, USA) equipped with UV diode array detection according to Olszewska (2007). Analysis of sugars (glucose, fructose, and sucrose) was performed by HPLC equipped with electrochemical detection (LC Bioinert 1260 Infinity, Decade elite ECD Antec.) based on the method of van Dam and Oomen (2008). Quantification of 104 samples was done by standard curves and expressed as μg g^–1^ leaf dry weight.

A 2,000 μg subsample of dried and ground tomato leaf material was weighted in tin foil cups for C and N content measurement using a FLASH 2000 organic elemental analyzer (Brechbuhler Incorporated, Interscience B.V., Breda, The Netherlands).

### 2.8. DNA extraction and soil microbiome sequencing

For fungal and bacterial community sequencing, DNA was extracted from 250 mg of rhizospheric soil using the PowerSoil DNA isolation kit (Qiagen, Hilden, Germany) as per the manufacturer’s instructions. For fungi, the intergenic transcribed spacer (ITS2) region was amplified using ITS4/ITS7 primers (5′-TCCTCCGCTTATTGATATGC-3′/5′- GTGAATCATCGAATCTTTG-3′) (Ihrmark et al., 2012), while for bacteria the V4 region was targeted using 515F/806R primers (5′-GTGYCAGCMGCCGCGGTAA-3′/5′- GGACTACNVGGGTWTCTAAT-3′) (Caporaso et al., 2012). The Amplicon sequencing was done using Illumina MISEQ PE with a 250 bp coverage. Library preparation and sequencing were done at Genome Quebec, Canada.

### 2.9. Statistical analysis

All statistical analyses were performed in R (R Core Team, 2021) and the graphs were created in R using the “ggplot2” package (Wickham, 2016). The number of spider mites 14 days after infestation was analyzed by a Poisson generalized linear mixed model (*log link function*) with fungal treatment (four categorical variables) as fixed effect and block as random factor. A linear mixed model was fitted to plant biomass and chemical data with fungal treatment, presence/absence of spider mites and their interaction as fixed effects and block as a random factor. A binomial generalized linear mixed effect model *(logit link function)* was fitted to the endophytic colonization data (presence/absence per plant piece and per plant tissue) using fungal treatment and plant tissue (stem and root) as fixed effects and block and plant id as random effects. Percentages of fungal colonization and confidence intervals were estimated based on the same models. Mixed models were fitted using the “lme4” package (Bates et al., 2015) and *P*-values were computed using the “lmerTest” package based on Satterthwaite’s approximation (Kuznetsova et al., 2017). Visual assessments of model fit were done by residual and quantile-quantile plots. The parameters with significant effects were subjected to pairwise comparisons using the Tukey *post-hoc* test by the “multcomp” package.

Fungal (ITS) and bacterial (16S) amplicon sequencing data was analyzed using DADA2 pipeline (Callahan et al., 2016). For bacteria, standard settings were used with SILVA as a database (v. 132). For fungi the length-based filtering was relaxed and minLen = 50, maxEE = c (2.2) was used. UNITE (release 10.5.2021; Nilsson et al., 2019) was used to identify fungal amplicon sequence variants (ASVs). Both datasets were filtered to contain 10,000–60,000 ASVs by removing samples with higher or lower read numbers. Total sum scaling (TSS) was used to normalize the data and only fungi were included in the ITS dataset and only bacteria in the 16S dataset. Both time points were analyzed separately. Permutational multivariate ANOVA (PerMANOVA) models were created using Bray-Curtis distances in Vegan (adonis2; Oksanen et al., 2020). For T1 the model had only the fungal treatment as a factor, but for T2 both fungal treatment, spider mite infestation and their interaction were analyzed. Due to the dominance of *Metarhizium* in fungus-inoculated samples, another model for analysis of ITS data was created excluding *Metarhizium*. This restricted dataset was analyzed in the same way as the total dataset. DESeq2 was used to investigate the effect of the treatments on fungal genera and bacterial classes using 0.01 alpha and log2 fold change.

## 3. Results

### 3.1. Effects of *M. brunneum* and inoculation method on population growth of spider mites

**FIGURE 1:**
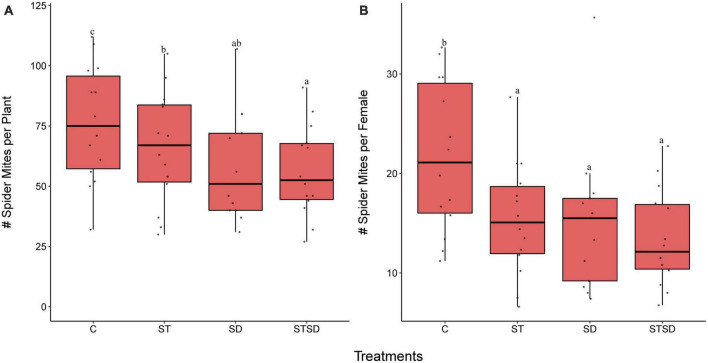
Population growth of two spotted spider mites (*Tetranychus urticae*) on tomato plants inoculated with *Metarhizium brunneum* as seed treatment (ST), soil drenching (SD), combination of seed treatment and soil drenching (STSD) and Triton X-100 control (C). Panel **(A)** shows the number of spider mites (juvenile and adults) per plant and panel **(B)** shows the number of spider mites per female after 14 days of infestation. Boxplots with different letters are significantly different at α = 0.05 within panels (by *post-hoc* test using multcomp function in R). The median is presented by a thick horizontal line in each box.

The number of spider mites after 14 days of infestation was significantly affected by *M. brunneum* inoculation (*P* < 0.001). All *M. brunneum* inoculations decreased the population size of spider mites compared to those on control plants (Figure 1A). However, plants inoculated with a combination of seed treatment and soil drenching showed significantly lower numbers of mites than plants that only received the seed treatment, whereas these numbers were not lower than those on plants that had only received the soil-drenching treatment (Figure 1A). The number of spider mites produced per founding female was also significantly lower (*P* < 0.0001) on *M. brunneum-*inoculated plants than on control plants, whereas no differences were observed among different fungal inoculation methods (Figure 1B).

### 3.2. Effects of *M. brunneum*, inoculation method and spider mite infestation on tomato plant growth

**FIGURE 2:**
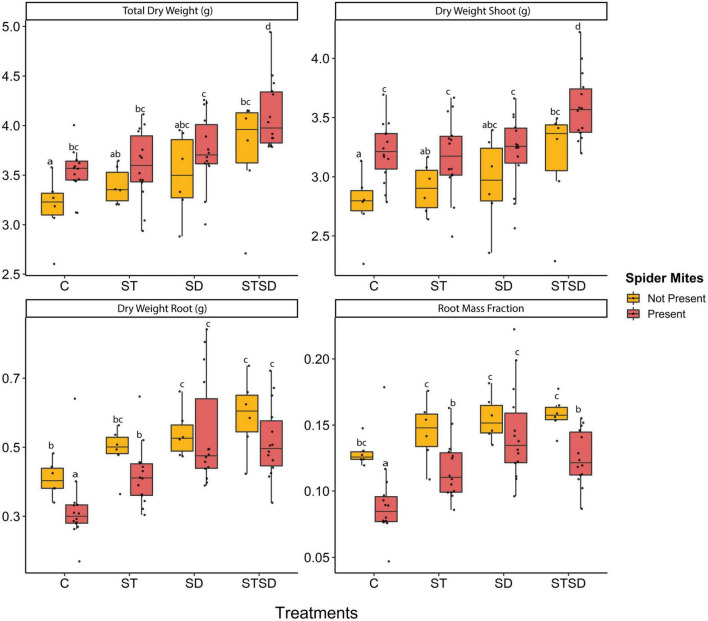
Growth parameters including total, shoot and root dry weight and root mass fraction of tomato plants inoculated with *Metarhizium brunneum* as seed treatment (ST), soil drenching (SD), combination of seed treatment and soil drenching (STSD) and Triton X-100 control (C) in the absence (yellow boxes) and presence (red boxes) of two spotted spider mites (*Tetranychus urticae*). Boxplots with different letters are significantly different at α = 0.05 within panels (by *post-hoc* test using multcomp function in R). The median is presented by a thick horizontal line in each box.

At T1, there was no significant effect of inoculation method on any of the growth measurements. However, at T2, both inoculation treatment and spider mite infestation significantly affected total, shoot and root dry weight as well as the proportion of biomass in roots (root mass fraction) (Supplementary Table 1). The total dry weight of tomato plants was increased both by inoculation with *M. brunneum* and by infestation with spider mites. The highest biomass was observed for plants inoculated by a combination of seed treatment and soil drenching in the presence of spider mites (Figure 2). Effects of inoculation treatment and spider mite infestation on plant dry weight slightly differed between roots and shoots. Plants subjected to a combination treatment of seed inoculation and soil drenching produced a significantly higher shoot dry biomass than plants subjected to either the single inoculation methods or control plants. However, plants subjected to soil drenching produced higher root dry weight than control plants, regardless of whether in addition they had received a seed inoculation treatment or not (Figure 2). Spider mite infestation enhanced the shoot dry weight but reduced the root dry weight of plants that had not been inoculated with the fungus. As a result, also the root mass fraction (root dry weight/total dry weight) was decreased in the presence of spider mites except in plants that had received fungal inoculation through soil drenching (Figure 2).

### 3.3. Effects of *M. brunneum*, inoculation method, and spider mite infestation on chlorogenic acid, rutin, sugars and C:N ratio

**FIGURE 3:**
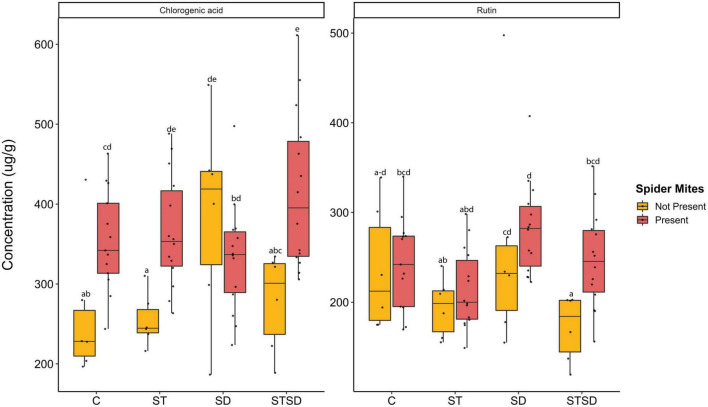
Levels of chlorogenic acid (CGA) and rutin (μg/g leaf dry weight) in tomato leaves inoculated with *Metarhizium brunneum* as seed treatment (ST), soil drenching (SD), combination of seed treatment and soil drenching (STSD) and Triton X-100 control (C) in the absence (yellow boxes) and presence (red boxes) of two spotted spider mites (*Tetranychus urticae*). Boxplots with different letters are significantly different at α = 0.05 within panels (by *post-hoc* test using multcomp function in R). The median is presented by a thick horizontal line in each box.

At T1, inoculation method did not affect leaf concentrations of primary metabolites (glucose, fructose, and sucrose) or secondary metabolites (chlorogenic acid and rutin). At T2, fungal treatments affected the levels of rutin and CGA, but the effects of fungal inoculation on CGA depended on spider mite infestation (Supplementary Table 2). Spider mites overall enhanced levels of CGA, except in plants that were inoculated with *M. brunneum* by soil drenching (Figure 3). In the absence of spider mites, *M. brunneum* enhanced levels of CGA compared to control plants when applied as soil drenching (Figure 3). In the presence of spider mites, *M. brunneum* also enhanced CGA levels compared to controls, but only when applied as a combination of seed inoculation and soil drenching. Under these conditions, *M. brunneum* thus reinforced the induction of CGA levels in response to spider mites. However, overall, there was no correlation between leaf CGA levels and the number of spider mites produced per female spider mite (*r* = +0.06, *n* = 55, *P* = 0.64).

Fungal treatments and spider mite feeding significantly and independently affected the leaf concentration of rutin (Supplementary Table 2). Spider mite infestation resulted in an overall increase in leaf rutin levels across all *M. brunneum* treatments (Figure 3). Leaf rutin levels did not differ between *M. brunneum* and control plants but differed between plants inoculated with different inoculation methods. In particular, in the absence of spider mites, levels of rutin were higher in plants inoculated by soil drenching than in plants inoculated by seed inoculation alone or by a combination of seed inoculation and soil drenching (Figure 3). However, as observed for CGA, there was no overall correlation between leaf levels of rutin and the number of spider mites produced per female spider mite (*r* = −0.04, *n* = 55, *P* = 0.77).

**FIGURE 4:**
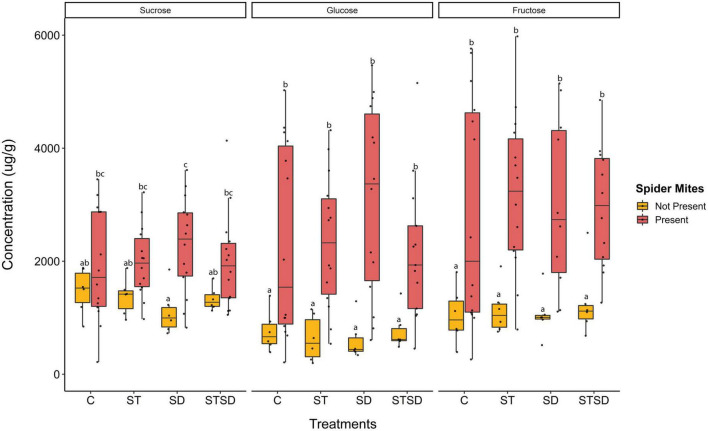
Levels of sugars (sucrose, glucose, and fructose) (μg/g leaf dry weight) in tomato leaves inoculated with *Metarhizium brunneum* as seed treatment (ST), soil drenching (SD), combination of seed treatment and soil drenching (STSD) and Triton X-100 control (C) in the absence (yellow boxes) and presence (red boxes) of two spotted spider mites (*Tetranychus urticae*). Boxplots with different letters are significantly different at α = 0.05 within panels (by *post-hoc* test using multcomp function in R). The median is presented by a thick horizontal line in each box.

The concentrations of the three main sugars (sucrose, glucose, and fructose) were significantly increased by spider mite feeding, however, they were not affected by fungal treatments or interactions between spider mite and fungal treatments (Supplementary Table 2). Sucrose levels increased by 30 to more than 100% in the presence of spider mites while levels of glucose and fructose increased even three to fivefold (Figure 4).

Leaf C:N ratio at T2 was also significantly affected by spider mite infestation but not by fungal treatment or interaction effects (Supplementary Figure 2; Supplementary Table 2).

### 3.4. Effects of different *M. brunneum* inoculation methods on soil microbial communities

**FIGURE 5:**
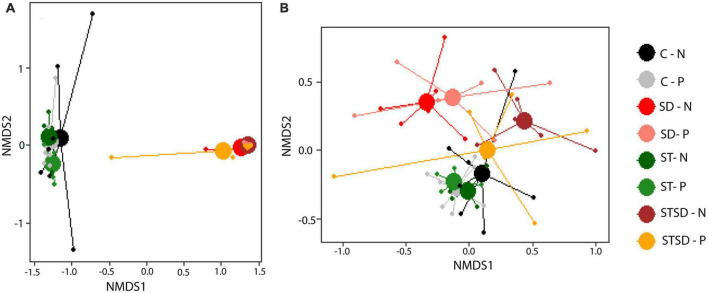
Non-metric multidimensional scaling (NMDS) plots of fungal communities (using Bray-Curtis distance) in the rhizosphere of tomato plants inoculated with *Metarhizium brunneum* as seed treatment (ST, green), soil drenching (SD, red), combination of seed treatment and soil drenching (STSD, orange/brown), or Triton X-100 control (C, gray/black) in the absence (N, light colors) and presence (P, dark colors) of two spotted spider mites (*Tetranychus urticae*). **(A)** Fungal communities including *Metarhizium* sp.; **(B)** fungal communities excluding *Metarhizium* sp. 2D stress for panel **(A)** = 0.08 and for panel **(B)** = 0.18. Treatment combination are indicated by a combination of the abbreviation for inoculation treatment (C, ST, SD, and STSD) and absence (N) or presence (P) of spider mites.

Inoculation with *M. brunneum* had a large impact on the composition of the rhizosphere fungal community. Multivariate analyses at T2 revealed that rhizosphere fungal communities of control and seed-inoculated plants differed significantly from plants with soil drenching treatment, either alone or in combination with seed treatment (*R*^2^ = 0.59; pseudo-*F* = 21.11, *P* < 0.001, Figure 5A), whereas these communities were not affected by spider mite infestation (*R*^2^ = 0.00; pseudo-*F* = 0.98, *P* = 0.325, Figure 5A). Not surprisingly, effects of inoculation on fungal communities were partly due to the high absolute (>2.5 × 10^5^) and relative abundance (mean = 0.78) of ASVs belonging to *Metarhizium* sp. (the added fungus) in the rhizosphere of soil-drenched plants compared to those of seed-treated (0.15 × 10^5^, 0.27) or control plants (0, 0.00) (Supplementary Figure 3). This resulted in a strong dominance of *Sordariomycetes* (the class of Ascomycota to which *M. brunneum* belongs) in the rhizosphere of soil-drenched plants (Supplementary Figure 3). More interestingly, even after removing ASVs belonging to the genus *Metarhizium* from the analysis, the composition of the resident fungal community still significantly differed between inoculation treatments without being affected by spider mite treatment (*R*^2^ = 0.15; pseudo-F = 2.61, *P* < 0.001; Figure 5B; Supplementary Figure 4). This indicates that soil inoculation also altered the relative abundances of fungi in the native community pool. Whereas resident fungal communities from the rhizosphere of control and seed treatment plants largely overlapped, they differed from those of soil-drenched plants (Figure 5B). Unexpectedly, resident fungal communities from the rhizosphere of plants that had only received soil drenching differed more from those of control plants than communities from plants that had received both soil drenching and seed treatment (Figure 5B). Closer inspection using DESeq2 revealed that in particular ASVs from the genus *Fusarium* had an increased abundance in the rhizosphere of plants that had been inoculated through soil drenching compared to control treatment. The effects of inoculation treatment on fungal community composition were already observed at T1 (*R*^2^ = 0.56, pseudo-F = 7.19, *P* < 0.001), but at that time point, the impact on the resident fungal community (excluding *Metarhizium*) was not yet observed (*R*^2^ = 0.12, pseudo-F = 0.93, *P* = 0.999).

Inoculation with *M. brunneum* not only affected the composition but also the diversity of the rhizosphere fungal community. The Simpson’s diversity index, which considers both the number of species and their relative abundances, of the rhizosphere fungal community was significantly reduced by inoculation through soil drenching, either alone or in combination with seed inoculation. This effect was maintained after removing *Metarhizium* ASVs from the analysis (Supplementary Figures 5A, B). At T1, effects of fungal inoculation on fungal community composition and diversity were similar to those at T2.

**FIGURE 6:**
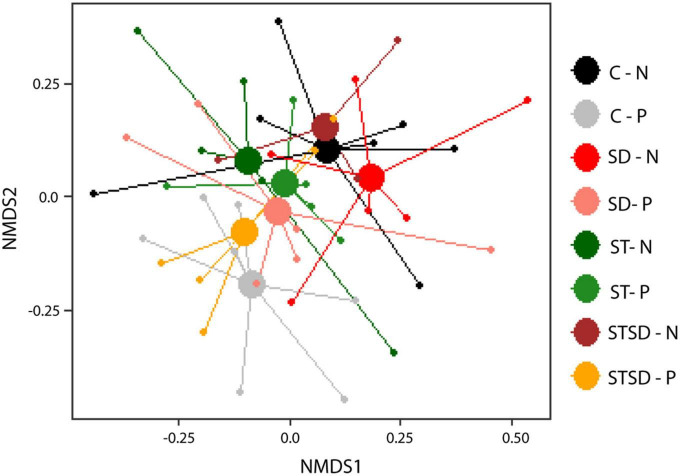
Non-metric multidimensional scaling (NMDS) plots of bacterial communities (using Bray–Curtis distance) in the rhizosphere of tomato plants inoculated with *Metarhizium brunneum* as seed treatment (ST, green), soil drenching (SD, red), combination of seed treatment and soil drenching (STSD, orange/brown), or Triton X-100 control (C, gray/black) in the absence (light colors) and presence (dark colors) of two-spotted spider mites (*Tetranychus urticae*). 2D stress = 0.14. Treatment combination are indicated by a combination of the abbreviation for inoculation treatment (C, ST, SD, and STSD) and absence (N) or presence (P) of spider mites.

In contrast to what was observed for the rhizosphere fungal communities, the composition (*R*^2^ = 0.07, pseudo-F = 1.01, *P* = 0.404; Figure 6) and diversity (Supplementary Figure 5C) of rhizosphere bacterial communities were not affected by inoculation with *M. brunneum*. However, effects of spider mite infestation on bacterial community composition were marginally significant (*R*^2^ = 0.02, pseudo-F = 1.03, *P* = 0.072; Figure 6). When only the control and the soil drenching treatment were included in the PerMANOVA model, we observed that spider mite infestation affected the bacterial community composition (*R*^2^ = 0.04, pseudo-F = 1.03, *P* = 0.041). The diversity of the rhizosphere bacterial community was not affected by spider mite infestation of plants (Supplementary Figure 5C). We further investigated which bacterial ASVs were affected by spider mite infestation. In total, 321 ASVs were significantly different between the spider mite treatments, and these belonged to 9 major phyla. Seven of these phyla contained both ASVs that were more abundant (175 ASVs) and ASVs that were less abundant in samples with spider mites (126 ASVs). By contrast, ASVs belonging to *Cyanobacteria* and *Planctomycetes* were only found to be more abundant in treatments with spider mites. On Phylum and Order level, we found more *Acidobacteria*, *Proteobacteria*, and *Firmicutes* (Bacillales) in the rhizosphere soils when spider mites were present (Supplementary Figure 6). As observed for the later timepoint T2 at which plants with and without spider mites were present (described above), also at the earlier timepoint T1, when only uninfested plants were studied, the bacterial composition was not affected by inoculation treatment (*R*^2^ = 0.14, pseudo-F = 0.99, *P* = 0.99).

### 3.5. Endophytic colonization with different *M. brunneum* inoculation methods

At T1, the proportion of root and stem pieces that were colonized by the fungus significantly differed between fungal treatments but not between tissues (Supplementary Table 3). Plants inoculated by seed inoculation showed no colonization in stems and very low colonization in roots. By contrast, plants inoculated by soil drenching showed higher colonization in the stem and roots than plants inoculated by the combination treatment (Table 1). At T2, a subset of the plants was infested with spider mites. Interestingly, at this time point, the presence of spider mites significantly reduced colonization levels of roots and stems, but fungal inoculation treatment no longer had a significant effect on colonization levels, and colonization levels again did not differ between roots and stem (Table 1; Supplementary Table 3). The level of root and stem colonization by *M. brunneum* was positively correlated with the shoot biomass of plants that were not infested by spider mites (*r* = +0.437, *n* = 24, *P* = 0.032 and *r* = +0.575, *n* = 24, *P* = 0.003, respectively), but these correlations were not observed in spider mite-infested plants (*r* = −0.022 and *r* = +0.139, *n* = 55, *P* > 0.3). The levels of root or stem colonization were not correlated with leaf concentrations of CGA or rutin in either infested or non-infested plants (all *P* > 0.07). None of the leaf sections tested was colonized by *M. brunneum.*

**TABLE 1:** Colonization of stems and roots of tomato plants by *Metarhizium brunneum* following inoculation as seed treatment (ST), soil drenching (SD), or a combination of seed treatment and soil drenching (STSD).

	Time point 1			Time point 2		
	Without spider mites		Without spider mites		With spider mites	
Treatments	Stem *n* = 36 (6 per plant)	Root *n* = 36 (6 per plant)	Stem *n* = 36 (6 per plant)	Root *n* = 36 (6 per plant)	Stem *n* = 84 (6 per plant)	Root *n* = 84 (6 per plant)
**Colonized tomato plant pieces % (95% CI)**						
ST	0% (0.0–13.7)a	3% (0.1–14.5)ab	14% (4.7–29.5)abc	17% (6.4–32.8)abc	5% (1.3–11.7)a	11% (5.0–19.4)abc
SD	44% (27.9–61.9)b	33% (18.6–51.0)b	14% (4.7–29.5)abc	19% (8.2–36.0)c	12% (5.9–20.8)abc	6% (2.0–13.3)ab
STSD	28% (14.2–45.2)b	25% (12.1–42.22)b	22% (10.1–39.2)c	17% (6.4–32.8)abc	15% (8.5–25.0)bc	18% (10.4–27.8)c

Values are percentages with 95% confidence intervals of the total number of colonized tomato tissue pieces at time point 1 (T1-4 weeks after seed treatment and 2 weeks after soil drenching) and time point 2 (T2- 6 weeks after seed treatment and 4 weeks after soil drenching). Note that at T1 all plants are without spider mites, whereas at T2 there are plants with and without spider mites. Same letters for colonized plant tissue pieces at time points 1 and 2, respectively, indicate no significant differences at α = 0.05 (by *post-hoc* tests using multcomp function).

## 4. Discussion

Since their discovery as plant endophytes, soilborne EPF such as *Metarhizium* spp., have been well exploited for their growth promotion and pest suppression effects after colonizing crop plants (Vega, 2018). Research on the endophytic abilities and growth promotion effects of *M. brunneum* has been more extensive than research on its potential for pest control as a plant endophyte. The current study is the first to report that using seed treatment, soil drenching, and a combination of both methods of inoculating tomato plants with *M. brunneum* was able to effectively reduce spider mite populations when compared to control plants.

Previous research has shown that direct application of various *Metarhizium* spp. can have lethal effects on spider mites (Castro et al., 2018; Ment et al., 2020). However, *M. brunneum* has been reported to both enhance and reduce pest populations in different crops as plant endophytes. Seed treatments with *M. brunneum* increased the population growth of aphids and spider mites in different crop hosts (Clifton et al., 2018; Rasool et al., 2021a,b). By contrast, soil drenching reduced the population growth of female spider mites in common bean (Ment et al., 2020), and colonization of melon plants by *M. brunneum* following foliar application caused mortality in *Spodoptera littoralis* (Garrido-Jurado et al., 2020). The efficacy of fungal applications to crops for pest management can be influenced by various factors, e.g., fungal isolate, growth substrate, plant host and other biotic and abiotic factors (Bamisile et al., 2021), which may lead to variability in the strength and direction of effects of *M. brunneum* in different studies. Moreover, the sub-lethal effects on spider mite populations in the present study are likely caused by indirect, plant-mediated defense responses, e.g., the production of specialized metabolites by the plant in response to fungal infection (Gange et al., 2019), rather than by direct effects of the endophytic fungi on the arthropods.

Various phenolic compounds, including flavonoids, play a vital role in plant tolerance to abiotic stresses as well as defense against biotic stresses, including arthropod herbivores (Simmonds, 2003). We found that mite-infested plants produced higher concentrations of both CGA and rutin than non-infested plants. This suggests that spider mites likely triggered an increase in the production of these secondary metabolites in plants as a defense mechanism, as plants are known to activate defense responses upon sensing herbivore attack (War et al., 2020). However, inoculation with *M. brunneum* via soil drenching also induced higher levels of CGA even in the absence of mites. In the presence of spider mites, the combination of seed treatment and soil drenching produced higher levels of CGA than observed in mock-inoculated plants, indicating that the fungus reinforced the plants’ response to spider mites, and under these conditions indeed the lowest number of spider mites were observed. However, we did not observe an overall negative correlation between leaf levels of CGA and spider mite population growth. Therefore, we cannot confirm a role of CGA in spider mite resistance in our experiment, and it is unclear whether the fungus-induced increase in this metabolite contributed to the observed fungus-induced spider mite resistance. Several other studies have shown that CGA can exert a broad spectrum of anti-herbivory activities either as a single compound or in combination with other plant metabolites (Liu et al., 2017; Kundu and Vadassery, 2019). A study showed that leaf CGA concentration is the main factor explaining variation in resistance against trips in chrysanthemum (Leiss et al., 2009). Metabolic engineering techniques have been applied to crops to increase the biosynthesis of these compounds to enhance crop functionalities (Mutha et al., 2021). Our study shows that one of the possible ways to achieve this is by applying an entomopathogenic fungus that can increase the production of these bioactive compounds in the crop. Several studies have suggested that a combination of several phenolic compounds, rather than a single compound, work simultaneously to protect against insects (Wink, 2003; Haukioja, 2006). Different inoculation methods may activate different sets of compounds in addition to CGA and rutin, therefore, we suggest exploring the whole metabolic profiles of treated plants to fully understand the underlying mechanisms.

Primary metabolites like sugars are involved in the growth and development of plants leading to increased plant tolerance against stress and also serving as precursors for the production of secondary metabolites (War et al., 2020). However, the role of sugars in herbivore pest defense is understudied, especially with respect to how this role might be modified by the presence of endophytic fungi. This study found that the leaf concentrations of three main sugars (sucrose, glucose, and fructose) were tremendously increased after spider mite infestation, while no effects of fungal inoculation treatments were found. Previously, the artificial application of sucrose has been shown to have a negative effect on insect population growth while glucose and fructose were shown to have a positive effect (Haukioja, 2006), however, we did not find such a trend. High sugar levels in infested leaves indicate that spider mite feeding impaired the export of sugars, probably to ensure a high carbohydrate content at the feeding site. The same behavior has been observed in Silverleaf white fly in cotton (Lin et al., 2000). Conversely, it can also be a part of plant responses to stress, for instance, during pathogen infection sugars interact with hormonal signaling and stimulate the biosynthesis of flavonoids for better plant immune responses (Morkunas and Ratajczak, 2014). There was a 30–100% increase in sucrose levels, while three- to fivefold increase in glucose and fructose levels was observed following spider mite feeding, which also suggests that spider mite infestation induced the production of invertases (Roitsch and González, 2004). Nevertheless, detailed studies should be conducted to test these hypotheses to decipher the role of sugars in the plant-herbivore interaction.

The ability of endophytic fungi to produce bioactive compounds can not only enhance plant stress tolerance but may also boost their growth by improving nutrient uptake (Bamisile et al., 2021). Previous research has demonstrated the potential of *M. brunneum* to enhance nutrient uptake (Behie and Bidochka, 2014b; Sánchez-Rodríguez et al., 2016) and increase both above- and below-ground biomass of various crops, under stressed and non-stressed conditions (Jaber and Enkerli, 2017; Rasool et al., 2021b). Although it has been emphasized that establishing a good inoculation method for better colonization and growth responses is crucial (Jaber and Enkerli, 2016), it has not been tested how various inoculation methods affect growth. This study showed that the combination of seed inoculation and soil drenching yielded the highest shoot biomass in both spider mite-infested and non-infested plants while the shoot biomass of plants inoculated through seed treatment or soil drenching alone did not significantly differ from the shoot biomass of control plants. Furthermore, all fungal inoculation methods resulted in increased root biomass compared to that of control plants in infested plants. Rasool et al. (2021b) also showed that seed inoculations of wheat and bean increased shoot and root biomass only in aphid-infested plants. The most prominent aspect of the growth response was that spider mite infestation enhanced shoot biomass but reduced root biomass. This indicates a trade-off between above and below-ground plant parts in response to foliar herbivores. Conversely, plants are shown to partition more biomass toward roots under poor nutrient and climatic conditions (Qi et al., 2019).

Our study shows that plant colonization rates by *M. brunneum* were strongly dependent on the inoculation method and were dynamic over time. At the initial time point, 4 weeks after seed treatment and 2 weeks after soil drenching, plants that had received a seed treatment did not show any colonization in the stem and only 3% in the root, while soil drenching and the combination treatment showed 25–45% in both root and stem. Effects of the inoculation method on endophytic colonization rates have been observed in various crop hosts (Bamisile et al., 2018). At the second time point, 6 weeks after seed inoculation and 4 weeks after soil drenching, the effect of inoculation method disappeared, but spider mite infestation significantly reduced *M. brunneum* colonization rates. Similar negative effects of aboveground herbivory on colonization by beneficial soil microbes have been observed for arbuscular mycorrhizal fungi (Barber et al., 2012). Interestingly, a positive correlation was observed between endophytic colonization rate and above-ground biomass in non-infested plants. Another study has shown that the extent of colonization of maize plants by *M. robertsii* was positively correlated with above-ground biomass and plant height (Ahmad et al., 2020), which suggests that endophytic fungi may facilitate nutrient transfer to above-ground plant parts (Behie and Bidochka, 2014a). In contrast to effects on aboveground plant biomass, colonization rates by *M. brunneum* were not correlated with spider mite population size, indicating that the spider mite resistance was induced by the presence of the fungus but not related to its extent of plant colonization.

Rhizosphere microbiota can not only influence plant growth and development but also increase resistance to pests, diseases and heavy metals (Morgan et al., 2005). However, the composition of microbial communities is sensitive to external disturbances (Allison and Martiny, 2008), which can influence their related ecological roles. For instance, the secretion of photosynthates through root exudates strongly affects rhizosphere microbial populations (Dai et al., 2021). But also the application of bio-inoculants, including fungi and bacteria, could affect soil microbial composition and diversity that ultimately influences plant performance and outcomes toward different ecological interactions (Cornell et al., 2021). Bacteria are known to respond faster to external factors than fungi. One limitation of our study is its short time duration, which makes it more difficult to detect effects on microbial diversity, especially since time is an important factor for changes in bacterial compositions (Hannula et al., 2019). The physiological and molecular factors involved in fungal rhizosphere competencies are not very well understood. Here we show, contrary to our expectation, that adding *Metarhizium*, especially when done in drenching, impacted the fungal community composition but not the bacterial community composition, which was only affected by the presence of spider mites. We speculate that the effects on fungi are through direct interaction with *Metarhizium* (making up to 80% of the relative abundance of the fungal community), leading to increased competition between species. When *Metarhizium* was less dominant in the community (through seed application), the effects on the community structure of fungi were less evident, indicating that there might be dose-dependency. The effects of spider mites were more evident for the bacterial than for the fungal community, but the role of the various bacteria that either increased or decreased in abundance due to spider mite presence remains to be tested. These changes are potentially due to direct interactions with spider mites, or due to changes in plant quality, root exudation patterns, changes in release of signaling compounds (Hu et al., 2018) or due to other mechanisms. In particular, an increasing number of studies has shown the importance of biotic stress-induced changes in root exudation patterns that lead to the recruitment of rhizosphere bacteria that subsequently contribute to enhanced plant immunity or the ability of the plant to cope with the biotic stress that triggered their recruitment (Berendsen et al., 2012, 2018; Hu et al., 2018; Carrión et al., 2019).

One explicit factor investigated in our study was the use of different methods for the application of *M. brunneum* in tomato plants. Some responses were strongly affected by differences in inoculation methods, e.g., growth and rhizosphere microbial communities, while others were less strongly affected, e.g., population growth of spider mites, levels of tested metabolites and rate of endophytic colonization. Interestingly, plant inoculation by soil drenching or seed inoculation alone led to less strong effects on growth, but not to less strong effects on spider mite resistance compared to the combination treatment. Likewise, soil drenching affected fungal microbial communities more than seed treatment, possibly due to the higher abundance of *M. brunneum* in the rhizosphere resulting from drenching compared to seed inoculation. On the one hand, it is critical to investigate in detail whether inoculation methods such as soil drenching that result in high rhizosphere densities of *M. brunneum* do not negatively affect the diversity or composition of rhizosphere fungal communities, on the other hand, the persistence of *Metarhizium* in high densities in the soil is considered good for biocontrol (Zimmermann, 2007). In any case, the inoculation method should be considered an important factor in studies with entomopathogens for a better understanding of plant responses and better use of these bio-control agents.

In conclusion, *M. brunneum* enhanced both tomato growth and tomato resistance to spider mites with all inoculation methods used, but treatments with soil drenching or a combination of seed treatment and soil drenching were more efficient. When applied as a combination of seed inoculation and soil drenching, *M. brunneum* reinforced the spider mite-induced production of CGA. Whereas such a priming effect could in principle contribute to the fungus-mediated reduction in spider mite population growth on these plants, we failed to observe an overall negative correlation between leaf CGA levels and spider mite population size, as observed for some other phloem and cell content feeders. We, therefore, speculate that fungal inoculation may have led to the production of other defense compounds that were not investigated in this study that were primarily responsible for, or acted synergistically with CGA, in activating fungus-induced plant defense. We further showed that inoculations with *M. brunneum*, especially through soil drenching, has a significant impact on the composition and diversity of the fungal but not the bacterial rhizosphere community. The consequences of such modulations need further study. We encourage further studies to focus on plant-mediated defense strategies of EPF for a more comprehensive understanding of mechanisms underlying their effects on pest resistance and growth promotion. Overall, the beneficial associations between EPF and plants represent an exciting area of research that could have important implications for sustainable agriculture and environmental management.

## Data availability statement

The sequencing data presented in this study is publicly available at the European Nucleotide Archive: https://www.ebi.ac.uk/ena: accession PRJEB61510 (ERP146602). Additional datasets generated and analyzed during the study are available from the corresponding author upon request.

## Author contributions

SR, AM, and AB conceived and designed the study. AM and SR performed the experiment, took samples, and processed the collected samples for chemical and molecular analysis. SH analyzed the molecular data for rhizosphere microbial communities. SR analyzed the rest of the data and wrote the original draft. SH and AB reviewed and edited/added substantially to the manuscript. All authors reviewed and approved the submission of our manuscript.
