# Supplementary material for: Effects of tomato inoculation with the entomopathogenic fungus Metarhizium brunneum on spider mite resistance and the rhizosphere microbial community
Source: Front Microbiol. 2023 May 24;14:1197770. doi: 10.3389/fmicb.2023.1197770 (PMC10244576; doi:10.3389/fmicb.2023.1197770)
Supplement: Supplementary file 1 [file Data_Sheet_1.docx]

Supplementary Material

Effects of tomato inoculation with the entomopathogenic fungus Metarhizium brunneum on spider mite resistance and the rhizosphere microbial community

Shumaila Rasool^l^, Andreas Markou^1^, S. Emilia Hannula^1,2^, Arjen Biere^1^

*** Correspondence:**Corresponding Author

S.Rasool@nioo.knaw.nl


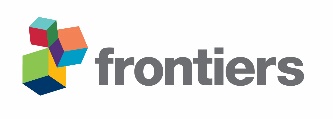


| **Supplementary Table 1.** Linear mixed models of the effects of fungal treatments, spider mite infestation and their interaction on growth characteristics of tomato plants, measured at two different time points at two time points (T1 and T2), 4 and 6 weeks after seed sowing, respectively. Note that at T1 all plants are without spider mites, whereas at T2 there are plants with and without spider mites. | | | | | | | | | | | |
| --- | --- | --- | --- | --- | --- | --- | --- | --- | --- | --- | --- |
|  |  |  | Total Dry Weight | | Dry Weight Shoot | | Dry Weight Root | | Root Mass Fraction | |  |
| Time points (T) | Effects | Df | F-ratio | P-value | F-ratio | P-value | F-ratio | P-value | F-ratio | P-value |  |
| T1 | Fungi | 3,20 | 0.496 | 0.68 | 0.70 | 0.56 | 2.84 | 0.063 | 3.88 | **0.030** |  |
| T2 | Fungi | 3,72 | 8.65 | **<0.0001** | 5.50 | **0.002** | 13.31 | **<0.001** | 9.29 | **<0.001** |  |
|  | Mites | 1,72 | 11.78 | **<0.001** | 23.32 | **<0.001** | 8.07 | **0.005** | 27.98 | **<0.001** |  |
|  | Fungi*Mites | 3,72 | 0.28 | 0.83 | 0.60 | 0.616 | 0.85 | 0.469 | 1.28 | 0.286 |  |
| Degrees of freedom (DF) columns show numerator degree of freedom (ndf) followed by denominator degree of freedom (ddf). Significant effects are highlighted in bold face. | | | | | | | | | | |  |

| **Supplementary Table 2.** Linear mixed models for the effects of fungal treatments, spider mites and their interaction on plant primary and secondary metabolites and C:N ratio measured at two different time points at two time points (T1 and T2), 4 and 6 weeks after seed sowing, respectively. Note that at T1 all plants are without spider mites, whereas at T2 there are plants with and without spider mites. | | | | | | | | | | | | | | | |
| --- | --- | --- | --- | --- | --- | --- | --- | --- | --- | --- | --- | --- | --- | --- | --- |
|  |  |  | Chlorogenic acid | | Rutin | | Sucrose | | Glucose | | Fructose | | C:N ratio | |  |
| Time points (T) | Effects | Df | F-ratio | P-value | F-ratio | P-value | F-ratio | P-value | F-ratio | P-value | F-ratio | P-value | F-ratio | P-value |  |
| T1 | Fungi | 3,20 | 1.12 | 0.364 | 1.14 | 0.353 | 0.63 | 0.077 | 0.93 | 0.441 | 0.36 | 0.777 | 0.22 | 0.878 |  |
| T2 | Fungi | 3,72 | 1.81 | 0.512 | 4.77 | **<0.01** | 0.004 | 0.999 | 0.22 | 0.875 | 0.44 | 0.719 | 0.67 | 0.575 |  |
|  | Mites | 1,72 | 13.81 | **<0.001** | 4.73 | **0.032** | 16.01 | **<0.001** | 31.98 | **<0.001** | 34.18 | **<0.001** | 4.72 | **0.037** |  |
|  | Fungi*Mites | 3,72 | 4.85 | **<0.01** | 1.12 | 0.344 | 0.76 | 0.518 | 0.40 | 0.747 | 0.40 | 0.750 | 0.89 | 0.455 |  |
| Degrees of freedom (DF) columns show numerator degree of freedom (ndf) followed by denominator degree of freedom (ddf). Significant effects are highlighted in bold face. | | | | | | | | | | | | | | | |

| **Supplementary Table 3.** Linear mixed models for the effects of fungal inoculation treatments, plant tissues (stem and root) and their interaction on endophytic colonization of tomato root and stem by *M. brunneum* at two different time points, 4 and 6 weeks after seed sowing, respectively. Note that at T1 all plants are without spider mites, whereas at T2 there are plants with and without spider mites. | | | | |
| --- | --- | --- | --- | --- |
| Time points (T) | Effects | Df | F-ratio | P-value |
| T1 | Fungi | 2,210 | 3.92 | **0.029** |
|  | Tissues | 1,210 | 0.52 | 0.467 |
|  | Fungi*Tissues | 2,210 | 0.04 | 0.954 |
| T2 | Fungi | 2,703 | 1.81 | 0.096 |
|  | Mites | 1,703 | 3.28 | **0.050** |
|  | Tissues | 1,703 | 0.09 | 0.834 |
|  | Fungi*Mites | 2,703 | 0.59 | 0.433 |
|  | Fungi*Tissues | 2,703 | 0.80 | 0.428 |
|  | Mites*Tissues | 1,703 | 0.06 | 0.798 |
|  | Fungi*Mites*Tissues | 2,703 | 1.45 | 0.225 |
| Degrees of freedom (DF) columns show numerator degree of freedom (ndf) followed by denominator degree of freedom (ddf). Significant effects are highlighted in bold face. | | | | |


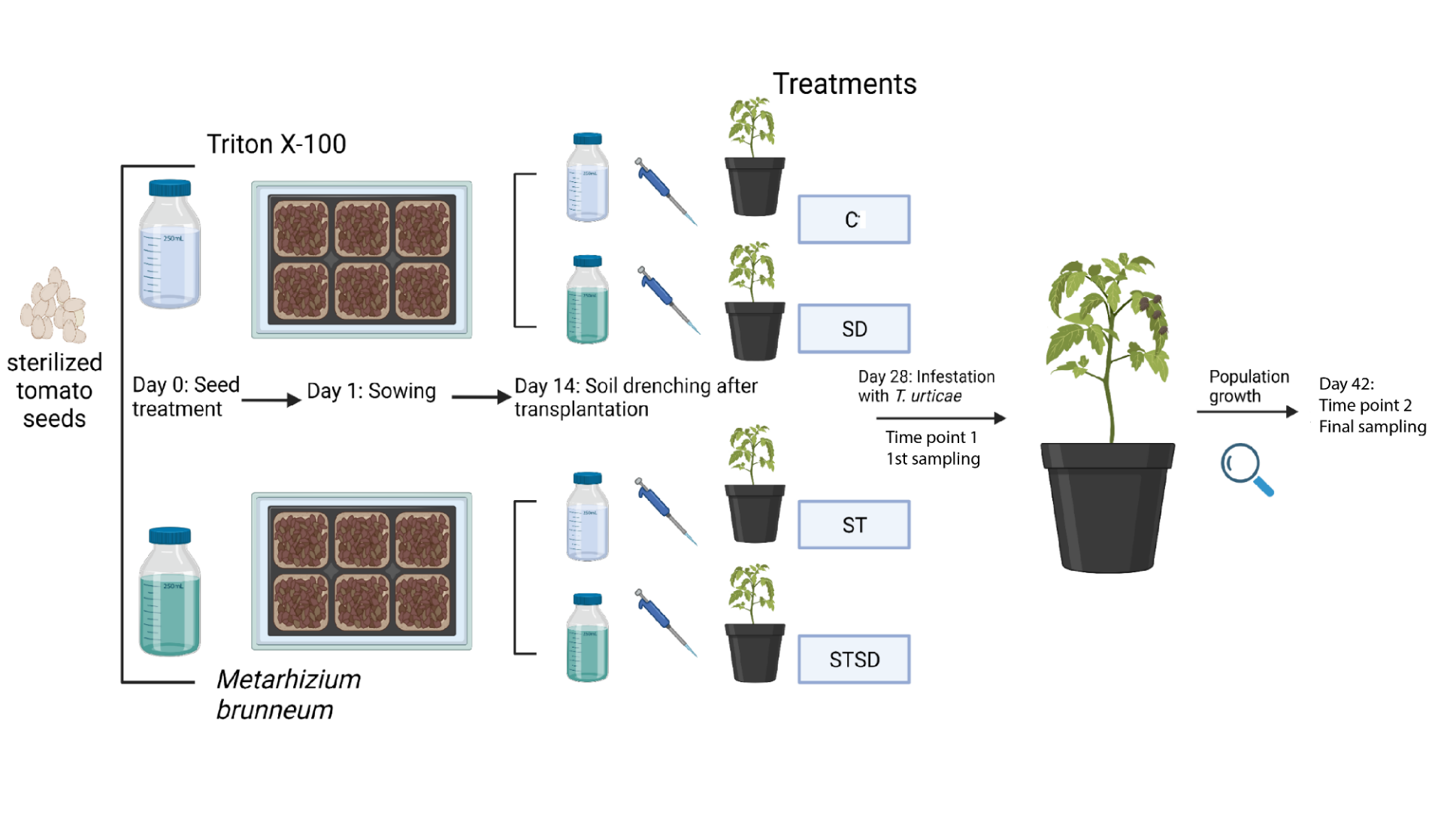


**Supplementary Figure 1** Schematic representation of experimental setup. Fungus (Metarhizium brunneum) and control (Triton X-100- C) treated sterilized tomato seeds were sown in germination trays. After two weeks, they were transplanted into individual pots and drenched with M. brunneum or Triton X-100, representing 3 fungal (seed treatment (ST), soil drenching (SD), combination of seed treatment and soil drenching (STSD) and a Triton X-100 control (C) treatment. The first sampling of 6 plants per treatment was done four weeks after seed treatment and two weeks after soil drenching (28 days) and two-spotted spider mites (Tetranychus urticae) were released on the rest of the plants (6 without mites and 14 with mites). Final sampling was done at six weeks after seed treatment and four weeks after soil drenching (42 days). This figure was created in BioRender ([biorender.com)](https://biorender.com/).


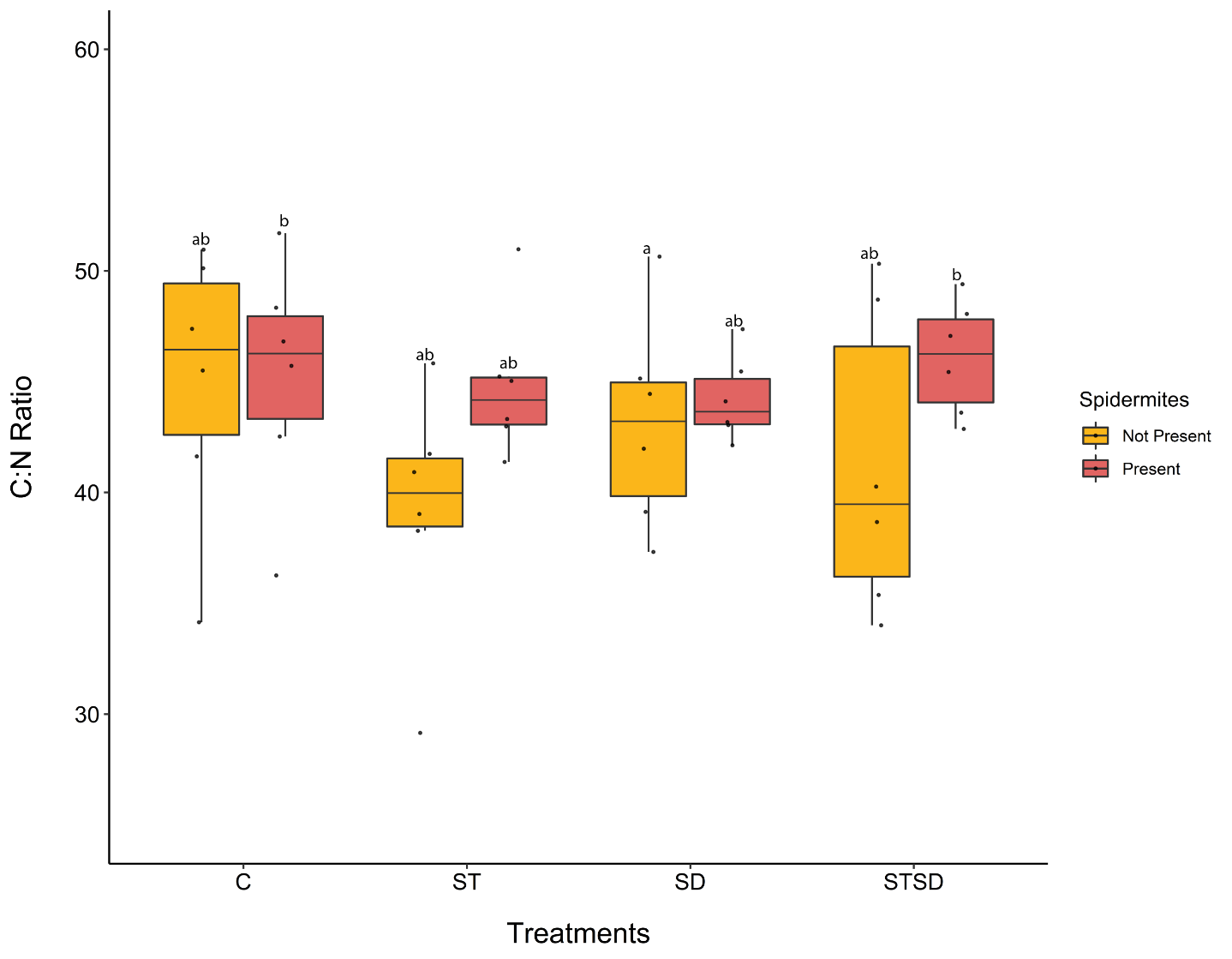


**Supplementary Figure 2:** Levels of C:N ratio in tomato leaves inoculated with *Metarhizium brunneum* as seed treatment (ST), soil drenching (SD), combination of seed treatment and soil drenching (STSD) and Triton X-100 control (C) in the absence (yellow boxes) and presence (red boxes) of two spotted spider mites (*Tetranychus urticae*). Boxplots with different letters are significantly different at α = 0.05 within panels (by post hoc test using multcomp function in R). The median is presented by a thick horizontal line in each box.


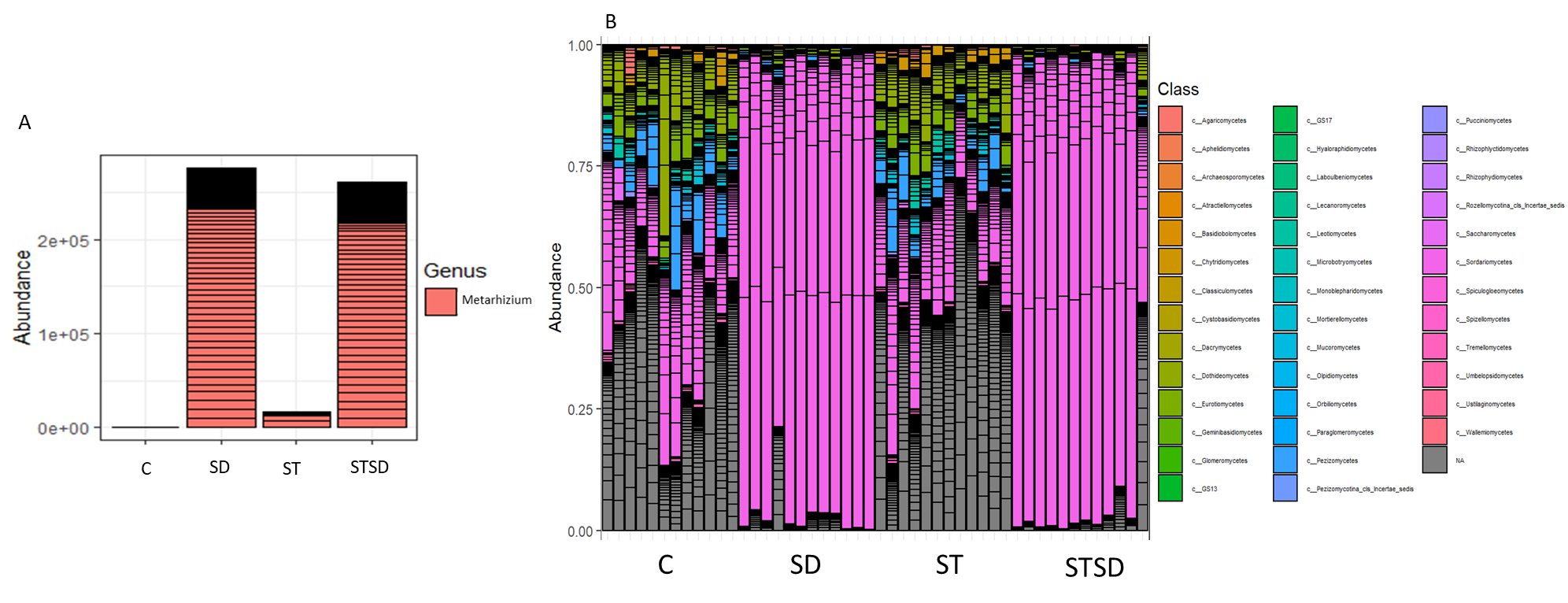


**Supplementary Figure 3**: A: Absolute abundance of *Metarhizium* sp. ASVs in the rhizosphere of tomato plants inoculated with *Metarhizium brunneum* as seed treatment (ST), soil drenching (SD), combination of seed treatment and soil drenching (STSD), or Triton X-100 control (C). B: Relative abundance of fungal classes in the rhizosphere of these plants (*n*=12 per fungal inoculation treatment).


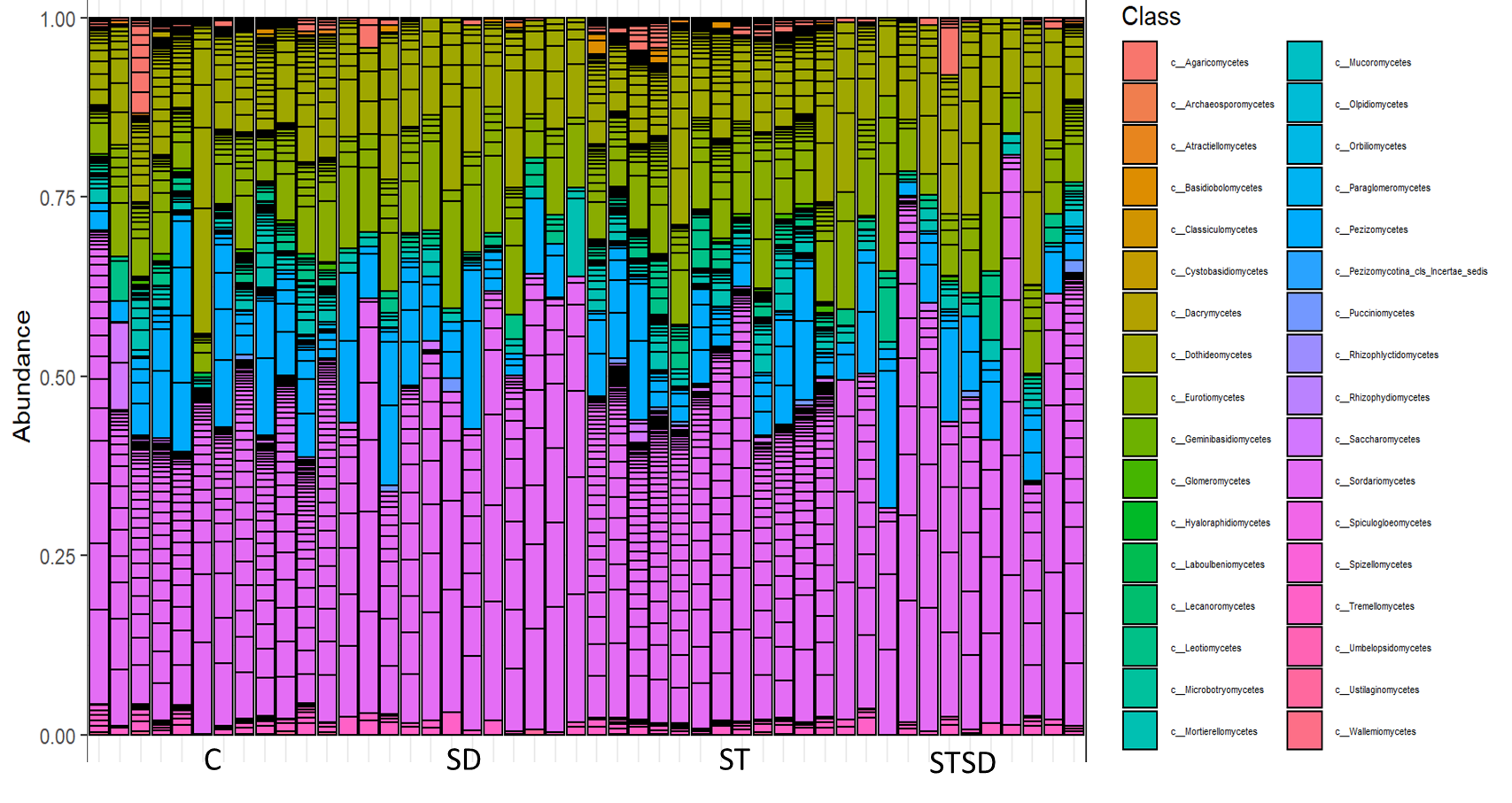


**Supplementary Figure 4**: Relative abundance of fungal classes in the rhizosphere of tomato plants inoculated with *Metarhizium brunneum* as seed treatment (ST), soil drenching (SD), combination of seed treatment and soil drenching (STSD), or Triton X-100 control (C) after removal of all *Metarhizium sp.* ASVs.

**Supplementary Figure 5**: A: Simpson’s diversity index of fungal ASVs in the rhizosphere of tomato plants inoculated with Metarhizium brunneum as seed treatment (ST), soil drenching (SD), combination of seed treatment and soil drenching (STSD), or Triton X-100 control (CT) in the absence (grey bars) or presence (black bars) of two spotted spider mites (Tetranychus urticae). B: Same after removal of all Metarhizium sp. ASVs. C: Simpson’s diversity index of bacterial ASVs.


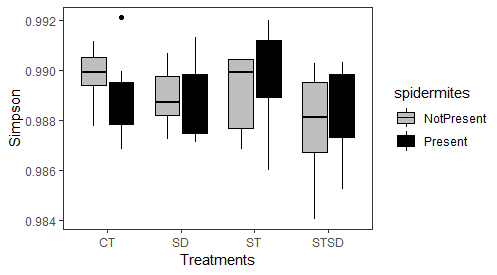

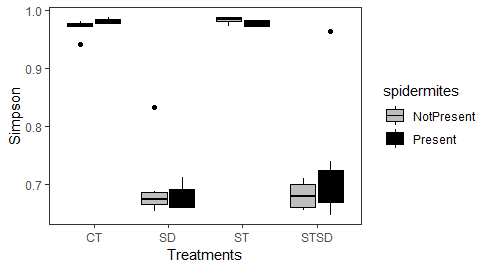

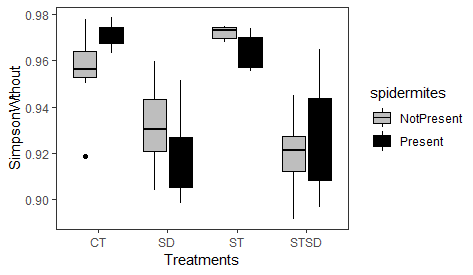


C

A

B


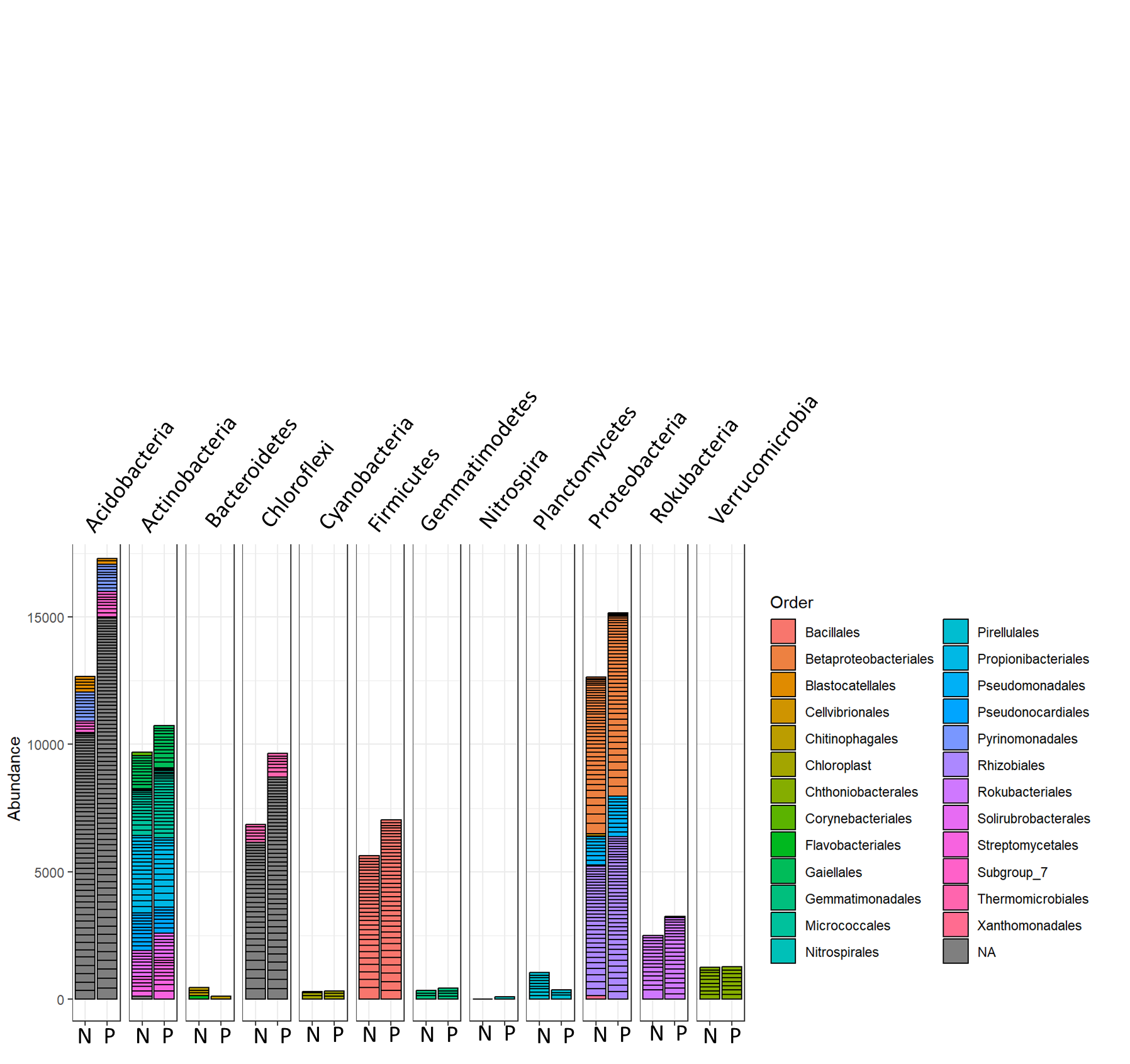


**Supplementary Figure 6**: Absolute abundance of bacterial orders in the rhizosphere of tomato plants across all fungal inoculation treatments that significantly differ in abundance in the absence (N) and presence (P) of two spotted spider mites (*Tetranychus urticae*).
